# Long-term predictive models of risk factors for early chronic kidney disease: a longitudinal study

**DOI:** 10.18632/oncotarget.24820

**Published:** 2018-04-13

**Authors:** Wen-Chih Wu, Po-Chien Hsieh, Fu-Kang Hu, Jen-Chun Kuan, Chi-Ming Chu, Chien-An Sun, Tsan Yang, Sui-Lung Su, Yu-Ching Chou

**Affiliations:** ^1^ School of Public Health, National Defense Medical Center, Taipei, Taiwan; ^2^ Department of Surgery, Suao and Yuanshan branches of Taipei Veterans General Hospital, Yilan, Taiwan; ^3^ Graduate Institute of Medical Sciences, National Defense Medical Center, Taipei, Taiwan; ^4^ Graduate Institute of Life Sciences, National Defense Medical Center, Taipei, Taiwan; ^5^ Department of Biostatistics, Firma Clinical Research, Hunt Valley, MD, USA; ^6^ Department of Public Health, College of Medicine, Fu-Jen Catholic University, New Taipei, Taiwan; ^7^ Big Data Research Center, College of Medicine, Fu-Jen Catholic University, New Taipei, Taiwan; ^8^ Department of Health Business Administration, Meiho University, Pingtung, Taiwan

**Keywords:** predictor, chronic kidney disease (CKD), aircrew, early detection, longitudinal study

## Abstract

**Background:**

The high incidence and prevalence of chronic kidney disease (CKD) in Taiwan have produced tremendous burdens on health care resources. The work environment of air force special operations personnel engenders high psychological stress, and the resulting increased blood pressure can lead to glomerular hypertension and accelerated glomerular injury in the long term. The aim of the study was to establish the predictive models to define the predictors of CKD.

**Results:**

The results indicated that the prevalence of CKD over 4 consecutive years was 3.8%, 9.4%, 9.0%, and 9.4%. The capability of using occult blood in urine to predict the risk of CKD after 1, 2, and 3 years was statistically significant. The age-adjusted odds ratio (OR) and 95% confidence interval (CI) were 7.94 (95% CI: 2.61–24.14), 12.35 (95% CI: 4.02–37.94) and 4.25 (95% CI: 1.32–13.70), respectively.

**Discussion:**

The predictive power of occult blood in urine for the risk of CKD in each model was statistically significant. Future investigations can explore the feasibility of implementing simple and accurate urine dipsticks for preliminary testing besides annual aircrew physical examinations to facilitate early detection and treatment.

**Methods:**

This study was a longitudinal study, in which air force special operations personnel who received physical examinations at military hospitals between 2004 and 2010 were selected. CKD was determined based on the definition provided by the US National Kidney Foundation. Overall, 212 participants that could be followed continuously for 4 years were analyzed.

## INTRODUCTION

Improvements in the medical care quality and dietary habit and lifestyle changes recently have led to the rising incidence and prevalence of chronic kidney disease (CKD) and end-stage renal disease (ESRD) worldwide. The high incidence and prevalence of ESRD in Taiwan is a concern requiring attention. The prevalence of early CKD is considerably higher than that of ESRD. CKD increases both all-cause mortality and medical expenditures [1]. Thus, CKD is a major disease that has become a focus in prevention and treatment efforts worldwide. The leading 10 causes of death in Taiwan have shifted from infectious diseases in the 1950s to primarily chronic degenerative diseases at present. Kidney disease (including nephritis, nephrotic syndrome, and nephrosis) was ranked 10th among Taiwan's leading causes of death for 2011, by the Department of Health, Executive Yuan.

CKD is the most prevalent chronic disease in Taiwan, accounting for 2.9% of the total mortality rate. The number of deaths related to this disease increased from the previous year [2]. CKD has been one of the leading 10 causes of death in Taiwan for more than 10 consecutive years. Air force work environments frequently subject aircrew to high psychological stress. This frequently causes hypertension in the long term. Increases in blood pressure can lead to glomerular hypertension, accelerated glomerular injury, and decreased renal function, thereby resulting in CKD [3]. Once CKD progresses into ESRD, most patients require kidney transplants or long-term dependence on dialysis to aid renal function. CKD also tends to be associated with complications such as hypertension, hyperlipidemia, diabetes, and cardiovascular disease [4, 5].

According to the results of the 2007 Follow-up Investigation on Hypertension, High Blood Glucose, and High Cholesterol in Taiwan by the Bureau of Health Promotion, the prevalence of CKD in Taiwan was 9.8% [6]. In 2008, Wen *et al*. conducted a large cohort study in Taiwan and found that the prevalence of stages 1 to 3 CKD was 11.6%, and the prevalence of stages 4 to 5 CKD was 0.3%. However, only 3.5% of the patients were aware that they had CKD [1]. These results indicate that CKD is a health problem in Taiwan and is also a crucial health issue that the Taiwanese military should consider. The aim of the study was to establish the predictive models to define the long-term predictions of risk factors for early CKD in Air Force Special Operations Personnel.

## RESULTS

### Basic demographic data

The study included 212 aircrew members in the air force. Analysis was performed on the results from the fourth physical examination (from the most recent year). The average age was 40.4 ± 7.15 years. Average height was 173.23 ± 5.52 cm. Average weight was 75.30 ± 8.47 kg. Average systolic blood pressure was 127.51 ± 7.60 mmHg and average diastolic blood pressure was 79.67 ± 6.31 mmHg.

### Previous physical examinations and CKD comparison

The results of 4 consecutive years of physical examinations from the valid sample of 212 aircrew members were analyzed and compared as shown in Table 1. The lowest average glomerular filtration rate (GFR) was 93.14 ± 16.01 mL/min/1.73 m2 as obtained from the fourth examination, whereas the highest average GFR was 107.29 ± 19.34 mL/min/1.73 m2 as obtained from the first examination. The prevalence of CKD was 3.8% (8 participants) at the first examination; 9.4% (20 participants) at the second examination; 9.0% (19 participants) at the third examination; and 9.4% (20 participants) at the fourth examination. The average GFR values and various groups exhibited significant statistical differences (*P* < .001) regarding the examination year.

**Table 1:** Comparison of previous examinations and CKD in the aircrew (*n* = 212)

Variable^a^	First examination		Second examination		Third examination		Fourth examination		*p* Value^b^
	*n* (%)	M ± SD	*n* (%)	M ± SD	*n* (%)	M ± SD	*n* (%)	M ± SD	
eGFR (ml/min/1.73 m^2^)		107.29 ± 19.34		103.58 ± 17.34		102.37 ± 16.84		93.14 ± 16.01	<0.001
≥90	171 (80.7)		164 (77.4)		161 (75.9)		119 (56.1)		<0.001
60-89	41 (19.3)		48 (22.6)		50 (23.6)		90 (42.5)		
45-59	0 (0)		0 (0)		1 (0.5)		3 (1.4)		
Presence of CKD									
non-CKD	204 (96.2)		192 (90.6)		193 (91.0)		192 (90.6)		0.084
CKD	8 (3.8)		20 (9.4)		19 (9.0)		20 (9.4)		
CKD stage									
Stage 1	8 (3.8)		14 (6.58)		11 (5.21)		10 (4.7)		0.136
Stage 2	0 (0)		6 (2.82)		7 (3.32)		7 (3.29)		
Stage 3	0 (0)		0 (0)		1 (0.47)		3 (1.41)		

^a^When the number of participants for each variable does not equal the total, this indicates missing values. The percentages are expressed as valid percentages.

^b^The *p* value for continuous variables is based on ANOVA, and that for categorical variables is the two-tailed significance level of the Chi-squared test.

M ± SD, Mean ± Standard deviation.

### Previous examination indicators and CKD risk comparison

Logistic regression analysis (Table 2) indicated that participants possessing abnormal urobilinogen levels were at a higher risk of CKD at each examination than participants possessing normal urobilinogen levels (*P* < .05). Those possessing abnormal urine ketone levels were at 4.88 times higher risk of CKD at the third examination (*P* < .05). Those possessing occult blood in urine were at a higher risk of CKD at the second, third, and fourth examinations (*P* < .01). Those possessing abnormal levels of white blood cells in urine were at 12.47 times higher risk of CKD at the second examination (*P* < .01). After controlling for age, participants demonstrating abnormal urobilinogen levels continued to be at a higher risk of CKD at each examination compared to those possessing normal urobilinogen levels (*P* < .05).

**Table 2:** Previous examination indicators and CKD risk comparison of aircrew (*n* = 212)

Variable	First examination		Second examination		Third examination		Fourth examination	
**OR (95% CI)**	**Adjusted OR (95% CI)^a^**	**OR (95% CI)**	**Adjusted OR (95% CI)^a^**	**OR (95% CI)**	**Adjusted OR (95% CI)^a^**	**OR (95% CI)**	**Adjusted OR (95% CI)^a^**
Age (year)	0.91 (0.80–1.03)		0.94 (0.87–1.01)		0.95 (0.89–1.03)		0.96 (0.90–1.03)	
Abnormal urobilinogen (vs normal)	19.80 (3.82–102.6)^**^	16.97 (3.16–91.19)^*^	6.07 (1.84–20.06)^*^	4.82 (1.36–17.05)^*^	8.26 (2.38–28.61)^*^	7.41 (2.09–26.29)^*^	4.59 (1.44–14.61)^*^	4.36 (1.36–13.99)^*^
Abnormal urine ketone (vs normal)	3.10 (0.34–27.91)	3.76 (0.40–35.80)	2.95 (0.88–9.95)	3.81 (1.07–13.60)^*^	4.88 (1.37–17.44)^*^	5.30 (1.45–19.42)^*^	1.22 (0.26–5.75)	1.35 (0.28–6.46)
Occult blood in urine (vs normal)	1.57 (0.18–13.54)	1.33 (0.15–11.58)	10.00 (3.44–29.11)^**^	8.71 (2.91–26.04)^**^	10.18 (3.68–28.15)^**^	9.54 (3.38–26.90)^**^	6.44 (2.33–17.83)^**^	6.11 (2.19–17.09)^*^
Abnormal white blood cells in the urine (vs normal)	N/A	N/A	12.47 (3.24–47.92)^**^	14.97 (3.64–61.58)^**^	2.40 (0.80–7.24)	3.39 (1.03–11.19)^*^	4.06 (0.98–16.74)	4.32 (1.03–18.09)^*^
High fasting blood glucose (vs normal)	1.93 (0.37–10.03)	3.14 (0.55–18.01)	1.04 (0.38–2.85)	1.33 (0.47–3.78)	0.56 (0.18–1.77)	0.66 (0.20–2.13)	0.30 (0.09–1.06)	0.32 (0.09–1.14)
Abnormal BUN (vs normal)	N/A	N/A	3.32 (0.33–33.47)	3.61 (0.35–37.21)	N/A	N/A	3.32 (0.33–33.47)	2.85 (0.27–29.71)
Abnormal serum creatinine (vs normal)	N/A	N/A	N/A	N/A	1.73 (0.20–15.19)	1.86 (0.21–16.53)	1.71 (0.47–6.39)	1.84 (0.47–6.99)
Abnormal uric acid (vs normal)	2.09 (0.06–8.62)	2.04 (0.49–8.50)	0.97 (0.34–2.82)	0.86 (0.30–2.53)	0.41 (0.11–1.45)	0.39 (0.11–1.40)	1.35 (0.46–3.95)	1.28 (0.43–3.76)
Abnormal LDL (vs normal)	5.50 (0.63–48.13)	7.89 (0.84–73.77)	2.27 (0.76–6.89)	2.13 (0.69–6.51)	1.01 (0.39–2.59)	1.13 (0.43–2.97)	0.42 (0.15–1.20)	0.44 (0.15–1.27)

^a^Adjustment for age.

OR, odds ratio; CI, confidence interval; N/A, not applicable; *p*< 0.05, *p*< 0.01

### Yearly CKD prediction analysis

The examination indicators from a specific year were used as independent variables and the presence of CKD in the subsequent first, second, and third years was used as the dependent variable. Logistic regression analysis (Tables 3 and 4) indicated that only occult blood in urine from a given year possessed predictive power for the risk of CKD in the subsequent year. Abnormalities in urobilinogen, occult blood in urine, white blood cells in the urine, and blood urea nitrogen (BUN) had predictive power for the risk of CKD in the second year following a particular year, although the OR of abnormal urobilinogen and that of abnormal BUN in Model 4 were not statistically significant. Abnormal urine ketone levels and occult blood in urine possessed predictive power for the risk of CKD in the third year following a particular year. The predictive power of occult blood in urine for the risk of CKD in each model was statistically significant.

**Table 3:** Aircrew physical examination indicators and CKD long-term predictions (*n* = 212)

Variable	Model 1		Model 2		Model 3	
	OR (95% CI)	Adjusted OR (95% CI)a	OR (95% CI)	Adjusted OR (95% CI)a	OR (95% CI)	Adjusted OR (95% CI)a
Abnormal urobilinogen (vs normal)	5.47 (1.26–23.85)^*^	4.77 (1.07–21.32)^*^	2.83 (0.72–11.07)	2.27 (0.54–9.44)	5.08 (1.41–18.35)^*^	4.62 (1.25–17.06)^*^
Abnormal urine ketone (vs normal)	1.07 (0.13–8.91)	1.18 (0.14–9.95)	1.22 (0.26–5.72)	1.41 (0.29–6.79)	1.67 (0.35–8.04)	1.74 (0.36–8.48)
Occult blood in urine (vs normal)	8.86 (2.94–26.67)^**^	7.94 (2.61–24.14)^**^	8.08 (2.72–24.00)^**^	7.37 (2.40–22.63)^**^	4.16 (1.50–11.55)^*^	3.88 (1.37–10.94)^*^
Abnormal white blood cells in urine (vs normal)	1.63 (0.19–14.28)	1.78 (0.20–16.01)	2.72 (0.54–13.85)	2.89 (0.56–14.95)	1.08 (0.30–3.93)	1.31 (0.34–5.03)
High fasting blood glucose (vs normal)	1.46 (0.46–4.70)	2.03 (0.59–6.97)	0.85 (0.29–2.48)	1.02 (0.34–3.06)	0.22 (0.05–0.99)^*^	0.24 (0.05–1.10)
Abnormal BUN (vs normal)	0.87 (0.11–7.09)	0.92 (0.11–7.63)	3.52 (0.35–35.60)	3.71 (0.36–38.02)	1.63 (0.19–14.28)	1.67 (0.19–14.71)
Abnormal serum creatinine (vs normal)	N/A	N/A	N/A	N/A	1.63 (0.19–14.28)	1.72 (0.20–15.18)
Abnormal uric acid (vs normal)	0.86 (0.32–2.34)	0.83 (0.30–2.28)	0.15 (0.02–1.13)	0.13 (0.02–1.01)	0.99 (0.36–2.70)	0.97 (0.35–2.65)
Abnormal LDL (vs normal)	1.87 (0.62–5.65)	1.52 (0.47–4.93)	1.09 (0.37–3.20)	1.04 (0.35–3.13)	0.48 (0.18–1.32)	0.51 (0.91–1.05)

^a^Adjustment for age.

Model 1: employed indicators from the first examination to predict the risk of CKD at the second examination.

Model 2: used indicators from the second examination to predict the risk of CKD at the third examination.

Model 3: adopted indicators from the third examination to predict the risk of CKD at the fourth examination.

OR, odds ratio; CI, confidence interval; N/A, not applicable; ^*^P < 0.05, ^**^P < 0.01

**Table 4:** Aircrew examination indicators and CKD long-term predictions (*n* = 212)

Variable	Model 4		Model 5		Model 6	
	OR (95% CI)	Adjusted OR (95% CI)a	OR (95% CI)	Adjusted OR (95% CI)a	OR (95% CI)	Adjusted OR (95% CI)a
Abnormal urobilinogen (vs normal)	3.13 (0.60–16.25)	2.77 (0.52–14.68)	1.21 (0.14–10.21)	1.08 (0.13–9.25)	6.07 (1.84–20.06)^*^	5.61 (1.57–20.09)^*^
Abnormal urine ketone (vs normal)	2.27 (0.54–13.85)	2.95 (0.57–15.25)	4.66 (1.10–19.70)^*^	5.00 (1.17–21.50)^*^	2.95 (0.88–9.95)	3.44 (0.99–12.04)
Occult blood in urine (vs normal)	13.31 (4.38–40.43)^**^	12.35 (4.02–37.94)^**^	4.59 (1.44–14.61)^*^	4.25 (1.32–13.70)^*^	3.93 (1.26–12.31)^*^	3.57 (1.11–11.50)^*^
Abnormal white blood cells in urine (vs normal)	N/A	N/A	1.63 (0.19–14.28)	1.72 (0.20–15.26)	4.66 (1.10–19.70)^*^	4.93 (1.15–21.14)^*^
High fasting blood glucose (vs normal)	0.64 (0.14–2.91)	0.78 (0.17–3.67)	0.60 (0.13–2.72)	0.70 (0.15–3.27)	0.58 (0.19–1.80)	0.65 (0.20–2.09)
Abnormal BUN (vs normal)	0.92 (0.11–7.53)	0.96 (0.12–7.95)	3.59 (0.89–14.52)	3.78 (0.92–15.49)	10.56 (1.40–79.47)^*^	11.07 (1.45–84.37)^*^
Abnormal serum creatinine (vs normal)	N/A	N/A	2.26 (0.45–11.27)	2.41 (0.48–12.17)	5.00 (0.43–57.73)	5.34 (0.45–63.10)
Abnormal uric acid (vs normal)	0.35 (0.10–1.25)	0.34 (0.10–1.22)	0.86 (0.32–2.34)	0.84 (0.31–2.29)	1.03 (0.36–2.98)	0.90 (0.31–2.63)
Abnormal LDL (vs normal)	1.21 (0.40–3.60)	1.05 (0.33–3.37)	1.46 (0.47–4.52)	1.12 (0.33–3.79)	2.50 (0.78–8.06)	2.31 (0.71–7.56)

^a^Adjustment for age.

Model 4: used indicators from the first examination to predict the risk of CKD at the third examination.

Model 5: employed indicators from the first examination to predict the risk of CKD at the fourth examination.

Model 6: adopted indicators from the second examination to predict the risk of CKD at the fourth examination.

OR, odds ratio; CI, confidence interval; N/A, not applicable; ^*^P < 0.05, ^**^P < 0.01

## DISCUSSION

According to the 2012 annual report from the US Renal Data System, the incidence of ESRD in Taiwan for 2010 was 361 per 1 million people, second to only the United States at 369 per 1 million people. However, the incidence has decreased gradually recently. The prevalence of ESRD in Taiwan was 2584 per 1 million people, which was the highest rate in the world [7]. Dialysis treatment is required when CKD advances into ESRD. In 2010, dialysis expenditures reached $1 billion. The Health Promotion Administration estimated that the annual growth rate in dialysis patients was approximately 6%. Data from the Taiwan Society of Nephrology indicated that 63,999 patients received dialysis treatment at the end of 2011, and the outpatient dialysis budget reached $1.1 billion. This was approximately 6% of the total health insurance budget [6, 8].

ESRD typically develops from CKD. Early CKD does not present clear symptoms, frequently resulting in delayed diagnosis and treatment. Therefore, highly sensitive, accurate, and ethnically appropriate screening methods should be established for the public and high-risk groups to facilitate early detection and treatment and to prevent the deterioration of renal function. Wen *et al*. performed a large cohort study in Taiwan, and used the aMDRD formula to estimate the prevalence of CKD at 11.93% (stages 1 to 5 were 1.02%, 3.79%, 6.81%, 0.22%, and 0.10%, respectively) [1]. This study also applied the aMDRD formula to obtain estimations, and the results indicated that the prevalence of CKD rose from 3.8% at the first examination to 9.4% at the fourth examination. The results also showed that although the overall prevalence was relatively low, the prevalence of stage 1 CKD at each examination was higher in the participants than that among the general population. This may be because aircrew members tend to be healthier than the general population, or because of aircrew receiving annual follow-up examinations and the effectiveness of early intervention by primary care teams. Thus, stage 1 CKD tended not to develop into stage 2. Abnormal GFRs and kidney damage may also occur simultaneously in early CKD patients. However, GFR decreases and renal pathological damage may appear alone, rather than concurrently [9]. This is because serum creatinine is secreted by renal tubules, and thus the initial response of serum creatinine to a GFR decrease is indistinct. The response increases only when renal function deteriorates to stage 3 CKD [10, 11]; that is, serum creatinine within the normal range does not necessarily indicate normal renal function.

Past studies have indicated numerous causes of CKD, the most common being hypertension and diabetes [12–16]. Hypertension is associated with CKD, patient age, degree of renal function decline, proteinuria, and primary kidney disease [17]. Hypertension is a crucial pathogenic factor of CKD. In CKD patients, hypertension influences the progression of kidney disease, accelerates the deterioration of renal function, and increases the risk of cardiovascular events [18]. Statistics have indicated that approximately 40% to 50% of new ESRD cases were caused by diabetes. The incidence of kidney disease caused by diabetes has doubled in the United States over the past 20 years, and the prevalence of CKD comorbid with both diabetes and hypertension has quadrupled [19]. Nevertheless, hypertension and high blood glucose (indicative of diabetes) did not exhibit statistically significant associations with CKD among the CKD risk factors examined in this study (table not shown). This may be because the blood pressure records in the collected data were insufficient (covering only 55 participants). In addition, high blood glucose might have been influenced by additional conditions (at least one laboratory result demonstrating urine protein, positive occult blood in urine, or a urine pH value greater than 8) of early CKD (stages 1 and 2), thereby preventing this factor from reaching statistical significance.

Currently, few studies have implemented long-term prediction analysis on the CKD risk factors for special populations. The importance of this study lies partly in verifying whether abnormal indicators in the health examinations of a particular year can be used to predict the risk of CKD in the subsequent first, second, and third years to enable early detection and treatment. The long-term prediction analysis indicated that occult blood in urine was statistically significant for predicting the risk of CKD in each model. The presence of red blood cells in the urine is referred to as occult blood in the urine. This is commonly used as a clinical indicator of kidney disease, such as kidney trauma, kidney stones, renal tumors, and acute nephritis. Although occult blood in urine does not indicate CKD directly, it is highly correlated with renal parenchymal damage [20]. Future research can examine the feasibility of implementing simple and accurate urine dipsticks for preliminary testing (eg, testing of occult blood in urine, urobilinogen, urine ketones, leukocyte esterase, urine protein, and urine pH value) besides annual aircrew physical examinations. This can facilitate early detection and treatment. The present study used the physical examination reports of 212 aircrew members from the Taiwanese military to perform a longitudinal study of predictors for early chronic kidney disease (CKD). The major findings of this study is that occult blood in urine is a statistically significant predictor of CKD risk. Identification of novel risk factors of CKD are important for better understanding, prevention and treatment of this disease. The longitudinal data used by this study come from a special population and are unique.

This study had some limitations. First, this study was unable to confirm whether the course of disease of renal parenchymal damage in early CKD, such as urine protein, positive occult blood in urine, or a urine pH value greater than 8, continued for more than 3 months. Second, air force aircrew members are screened rigorously because of the special operating environments of their work; only healthy personnel may continue to serve on missions. Therefore, cases of patients with stages 4 to 5 CKD were lacking. Consequently, caution is required when extrapolating the results of this study. Third, the physical examination data were generated by four hospitals, which may cause a batch effect in this study. However, the batch effect was small because all the aircrew members had the same protocol of physical examinations in Armed Forces General Hospital in Taiwan. Finally, since the requirements to aircrew members, who with diabetes or diseases will not be allowed to serve as an active aircrew, there were no participants who had been taking anti-hypertensive agents or anti-DM agents at the inclusion in the study. Furthermore, a significant drawback that hypertension is one of the most important factors leading to CKD. However, the contribution of hypertension to development of CKD could not be analyzed due to insufficient data (covering only 55 participants). We suggest a future work to follow up inactive aircrew members who with diseases that can incremental samples for investigating the contribution of hypertension to development of CKD.

In addition to calculating the GFR, the presence of renal parenchymal damage must also be considered to determine early CKD. This study indicated that occult blood in urine, urobilinogen, white blood cells in urine, and urine ketones were predictors for the future risk of CKD. Thus, the feasibility of implementing simple and accurate dipsticks for preliminary testing outside of annual aircrew physical examinations can be investigated in subsequent studies, conforming to the principles of “early detection, early treatment, and continued tracking.” Moreover, the confidence interval is wide due to small sample size. The interpretation of the result of this present study should be conservative, and further studies will be needed to confirm this findings.

## MATERIALS AND METHODS

### Study design

This study was a longitudinal study.

### Participants and data collection

Physical examination data were collected from 1398 aircrew members who received aircrew physical examinations between 2004 and 2010 at the following 4 military hospitals (or their branches): Taoyuan Armed Forces General Hospital, Taichung Armed Forces General Hospital, Kaohsiung Armed Forces General Hospital, and Hualien Armed Forces General Hospital. The study design and subject recruitment have been described elsewhere [21]. The data of 6 crewmembers were removed as duplicates and 235 samples were excluded because they contained incomplete data (data were considered incomplete if age, serum creatinine, urine protein, red blood cells in urine, or urine pH values were missing), and a final valid sample of 1157 aircrew members was obtained. The samples were participants who could be followed continuously for 4 years (212 male aircrew members). This study performed and examined long-term predictions of early CKD risk factors among the crewmembers. This study was exempted from full review and their written informed consents of participants, and approved by the Tri-Service General Hospital Institutional Review Board (TSGHIRB Approval Number 1-102-05-047) because the participants remained anonymous and the identification of participants had already encrypted, thereby complying with the Declaration of Helsinki without violation of participant privacy. The board is organized under, and operates per International Conference on Harmonization (ICH)/WHO Good Clinical Practice (GCP) and the applicable laws and regulations.

### Definition of CKD

In 2002, the National Kidney Foundation Kidney Disease Outcome Quality Initiative (NKF KOQI) has classified each stage of CKD based on GFR into 5 stages, defining the disease as follows [5]:

(1) Kidney damage for ≧3 months, as defined by structural or functional abnormalities of the kidney with or without decreased GFR, manifest by either: pathological abnormalities; or markers of kidney damage, including abnormalities in the composition of the blood or urine, or abnormalities in imaging tests.

(2) GFR < 60 mL/min/1.73 m^2^ for ≧3 months, with or without kidney damage.

In addition, stages 1 and 2 CKD must include one of the following: positive urine protein, positive occult blood in urine, or a urine pH value greater than 8.

### GFR formula

Numerous studies have developed formulas for convenient assessment and estimation of renal function. These formulas typically use serum creatinine to calculate the GFR. Because serum creatinine differs among individuals, the rate must be adjusted according to sex, age, and race [10]. This study used the abbreviated modification of diet in renal disease (aMDRD) formula that was revised by Levey *et al*. to calculate the GFR [22].

The aMDRD formula is as follows: estimated GFR = 186.3 × serum creatinine^−1.154^ × age^−0.203^ × 0.742 (if female) × 1.210 (if black). The unit is mL/min/1.73 m^2^.

### Statistical analysis

Descriptive results of continuous variables were expressed as mean (standard deviation), categorical variables were expressed as frequency (%). All statistical analyses were performed with one-way analysis of variance (ANOVA) and Chi-square tests. Multiple logistic regression was used to estimate odds ratios (ORs) and 95% confidence intervals (CIs) for the association between the potential risk factor levels and CKD risk. All statistical tests were two-tailed, and values of *p* < 0.05 were considered statistically significant. Data were analyzed using the IBM SPSS Statistics version 22 (IBM^®^ SPSS^®^ Statistics 22).

## Abbreviations

CKD: chronic kidney disease
MDRD: modification of diet in renal disease
GFR: glomerular filtration rate
OR: odds ratio
CI: confidence interval
ESRD: end-stage renal disease
TSGHIRB: Tri-Service General Hospital Institutional Review Board
NKF KOQI: National Kidney Foundation Kidney Disease Outcome Quality Initiative
ANOVA: analysis of variance.

## References

[1] Wen CP, Cheng TY, Tsai MK, Chang YC, Chan HT, Tsai SP, Chiang PH, Hsu CC, Sung PK, Hsu YH, Wen SF (2008). All-cause mortality attributable to chronic kidney disease: a prospective cohort study based on 462 293 adults in Taiwan. Lancet.

[2] Department of Health (2012). Cause of death statistics. Executive Yuan, ROC.

[3] Taal MW, Brenner BM (2006). Predicting initiation and progression of chronic kidney disease: developing renal risk scores. Kidney Int.

[4] Ma YC, Zuo L, Chen JH, Luo Q, Yu XQ, Li Y, Xu JS, Huang SM, Wang LN, Huang W, Wang M, Xu GB, Wang HY, Chinese eGFR Investigation Collaboration (2007). Improved GFR estimation by combined creatinine and cystatin C measurements. Kidney Int.

[5] National Kidney Foundation (2002). K/DOQI clinical practice guidelines for chronic kidney disease: evaluation, classification, and stratification. Am J Kidney Dis.

[6] Bureau of Health Promotion 2007 Follow-up Investigation on Hypertension, High Blood Glucose, and High Cholesterol (2008). Department of Health, ROC.

[7] U.S. Renal Data System and National Institute of Health (2012). USRDS Annual Data Report: International Comparisons. https://www.usrds.org/Default.aspx.

[8] National Health Insurance Administration and Ministry of Health and Welfare (2012). The National Health Insurance Statistics.

[9] Lee PH, Chang HY, Tung CW, Hsu YC, Lei CC, Chang HH, Shih YH, Lin CL (2009). Microalbuminuria, A Sign Usually Ignored. Journal of Internal Medicine of Taiwan.

[10] Stevens LA, Coresh J, Greene T, Levey AS (2006). Assessing kidney function—measured and estimated glomerular filtration rate. N Engl J Med.

[11] Artunc FH, Fischer IU, Risler T, Erley CM (2005). Improved estimation of GFR by serum cystatin C in patients undergoing cardiac catheterization. Int J Cardiol.

[12] Iseki K (2003). The okinawa screening program. J Am Soc Nephrol.

[13] Adler AI, Stevens RJ, Manley SE, Bilous RW, Cull CA, Holman RR, Ukpds G, UKPDS GROUP (2003). Development and progression of nephropathy in type 2 diabetes: the United Kingdom Prospective Diabetes Study (UKPDS 64). Kidney Int.

[14] Haroun MK, Jaar BG, Hoffman SC, Comstock GW, Klag MJ, Coresh J (2003). Risk factors for chronic kidney disease: a prospective study of 23,534 men and women in Washington County, Maryland. J Am Soc Nephrol.

[15] Fox CS, Larson MG, Leip EP, Culleton B, Wilson PW, Levy D (2004). Predictors of new-onset kidney disease in a community-based population. JAMA.

[16] Hwang SJ, Tsai JC, Chen HC (2010). Epidemiology, impact and preventive care of chronic kidney disease in Taiwan. Nephrology (Carlton).

[17] Ridao N, Luño J, García de Vinuesa S, Gómez F, Tejedor A, Valderrábano F (2001). Prevalence of hypertension in renal disease. Nephrol Dial Transplant.

[18] Toto RD (2005). Treatment of hypertension in chronic kidney disease. Semin Nephrol.

[19] Meguid El Nahas A, Bello AK (2005). Chronic kidney disease: the global challenge. Lancet.

[20] Bastos MG, Kirsztajn GM (2011). Chronic kidney disease: importance of early diagnosis, immediate referral and structured interdisciplinary approach to improve outcomes in patients not yet on dialysis. J Bras Nefrol.

[21] Chang HF, Tsai CD, Jhang GC, Lee MH, Chiu YL, Wu WC, Kuan JC, Lin LP, Chang SC, Chou YC (2015). Prevalence and factors associated with chronic kidney disease among military aircrews. J Med Sci.

[22] Levey AS, Greene T, Kusek JW, Beck GJ, Group MS (2000). A simplified equation to predict glomerular filtration rate from serum creatinine. J Am Soc Nephrol.

